# Supplementary material for: Performance of HIV rapid testing algorithm in Nigeria: Findings from a household-based Nigeria HIV/AIDS Indicator and Impact Survey (NAIIS)
Source: PLOS Glob Public Health. 2022 Jul 29;2(7):e0000466. doi: 10.1371/journal.pgph.0000466 (PMC10021238; doi:10.1371/journal.pgph.0000466)
Supplement: Attachment — Submitted filename: Response to Reviewers.docx [file pgph.0000466.s002.docx]

Response to Reviewers - Performance of HIV rapid testing algorithm in Nigeria: Findings from a household-based Nigeria HIV/AIDS Indicator and Impact Survey (NAIIS)

1. Please amend your detailed Financial Disclosure statement. This is published with the article, therefore should be completed in full sentences, and contain the exact wording you wish to be published.

State what role the funders took in the study. If the funders had no role in your study, please state: “The funders had no role in study design, data collection and analysis, decision to publish, or preparation of the manuscript.”

Response: Thank you for your observation. The Financial Disclosure statement is written as we wished to be published. We’ve added a statement about the role of the funders on page 2, lines 31 -32 of the tracked version of the manuscript: “*The funders had no role in study design, data collection and analysis, decision to publish, or preparation of the manuscript.”*

2. Please ensure that the funders and grant numbers match between the Financial Disclosure field and the Funding Information tab in your submission form. Note that the funders must be provided in the same order in both places as well.

We have verified the grant numbers and they match between the financial disclosure field and the funding information tab.

3. Please update your Competing Interests statement. If you have no competing interests to declare, please state: “The authors have declared that no competing interests exist.”

Updated and added language to the manuscript. Page 2, line 32, “*Competing Interests statement: The authors have declared that no competing interests exist.”*

4. Please provide a complete Data Availability Statement in the submission form, ensuring you include all necessary access information or a reason for why you are unable to make your data freely accessible. Note that it is not acceptable for the authors to be the sole named individuals responsible for ensuring data access.

PLOS defines a study's minimal data set as the underlying data used to reach the conclusions drawn in the manuscript and any additional data required to replicate the reported study findings in their entirety. Any potentially identifying patient information must be fully anonymized.

If your research concerns only data provided within your submission, please write “All data are in the manuscript and/or supporting information files” as your Data Availability Statement.

Justification: In general, these data cannot be shared publicly due to concerns about the risk of disclosing individual HIV status. However, we have provided the supplemental table (S1 File. Raw data for analysis) that was used for deriving the data presented in the manuscript.

5. Please provide separate figure files in .tif or .eps format only and ensure that all files are under our size limit of 20MB.

For more information about how to convert your figure files please see our guidelines: <https://journals.plos.org/globalpublichealth/s/figures>

 As requested, the figure has been converted to .tiff file and re-submitted online along with the requested revisions.

Comments to the reviewers: Authors would like to thank the reviewers for their constructive feedback and taking time out of their busy schedule in providing a thorough review. We think we have adequately addressed all your concerns raised in blue below.

**Reviewer #1: Comments for the Authors:**

1. Which of the test kits was the gold standard for determining the HIV status of the subjects?

Response - For the purposes of this study, Geenius HIV-1/2 supplemental assay and in some cases HIV-1 western blot assay, as needed, was used to confirm all HIV-positive reactivity as per Figure. Final status determined by outcome of these supplementary tests was used as the gold standard.

2. In this assessment why was sensitivity and negative predictive value not included?

Response - Since the study used the pre-established, Nigeria’s national HIV testing algorithm, all subjects tested by the first test, Determine HIV-1/2 rapid test, were concluded as negative if the results of this test were non-reactive. No additional testing was performed on the negative samples. In the manuscript we only report the positive predictive value of the national HIV testing algorithm since only the HIV-positive samples were further confirmed by the Geenius HIV-1/2 supplemental assay and WB.

Therefore, sensitivity of the algorithm was assumed 100%. No additional testing was conducted on the negative specimens. We have included the following language in the manuscript for additional clarification. Page 7, lines 141 and 142; “*The negative predictive value of the national testing algorithm was assumed to be 100%**. “*

3. What happened to the subjects that had false positive results? Was there any mechanism that recalled them for proper counseling?

Response - Thank you, for this important comment. Within the protocol, all results were critically reviewed and adjudicated in a case-by-case manner for follow-up and corrective action. In case of false positive, identified by the additional Geenius testing, were reported to ministry of health for further follow-up and review of the national testing algorithm.

4. The study had some limitations. One of them was that the national survey used some people without adequate training and experience with the test kits thus this could have contributed to the false positive cases. This should be stated. Recommendation should include to assess the performance of the test kits at health institutions where the actual testing and treatment take place.

Response: The study listed large trainings as one of the limitations, however multiple quality checks were built into monitoring of the study. We agree with your recommendation and to clarify lot-to-lot verification of all HIV rapid test kits were performed prior to field implementation. Additionally, quality control testing of first 50 HIV- rapid tests were performed for all testers to confirm their competency.

5. The conclusions and recommendations are not clear. This should be limited to the findings in the study.

Response: We believe we have clearly stated our conclusions and discussed our findings in the manuscript, presenting potential challenges of current national testing strategy that uses two consecutive positive tests in low prevalence settings. Furthermore, we have discussed pros and cons of two versus three testing strategies and discussed alternative ways to increase positive predictive value by retesting for verification as recommended by WHO. We believe Discussion offers additional opportunity to present our findings in the context of broader implications to HIV programs in the country. We believe manuscript covers it all well.

6. What are the authors recommending for HIV testing based on their findings?

Response: As discussed in the last section of the manuscript, Nigeria, and other countries with HIV prevalence <5% should consider moving to 3-test strategy to increase PPV of HIV diagnosis considering other local factors in the country. These recommendations are well articulated in the Discussion section with appropriate references to WHO guidance documents.

**Reviewer #2:**

This is an important paper describing the low PPV of the current 2-test HIV testing strategy employed in Nigeria, and as such it merits its publication for further dissemination. I commend the authors for sharing this important work and call for more accurate diagnosis of HIV to reduce the likelihood of misdiagnosis. I would advise authors not to be timid recommending a 3-test strategy even if retesting is in place, because this is currently the recommendation from WHO regardless of a 2-test or 3-test strategy and many countries are starting to take up this WHO recommendation. Please kindly see below some comments with the hope that this improves the current paper, it was very interesting to read this piece and I'm sure it will be of the interest of the readership of this journal.

Comments:

1. Lines 68–70: This paragraph needs revision because the latest WHO HIV testing guidelines in 2019 recommend that “three consecutive reactive tests should be used for an HIV-positive diagnosis” regardless of the HIV prevalence.

Response: Thank you, for your comment and per your recommendation we have updated the language to include the following: *“For HIV diagnosis, three consecutive reactive tests should be used for a HIV-positive diagnosis and must be reviewed …” (page 4, lines 69-70)*

2. Line 72–74: The authors state that false positive test results in an algorithm can be reduced by using a third test or confirmatory tests including Western Blot. Considering that the latest WHO HIV testing guidelines recommend countries to discontinue the use of Western Blot given its high rate of inconclusive results, which is evidenced in the paper with a WB indeterminate rate of 74.8% (98/131), I would suggest removing WB from this sentence.

Response: Thank you for your recommendation. Only HIV rapid testing is feasible at a health facility (non-lab setting). Testing with Geenius is recommended as a near point of care tests and not all facility have capacity to perform this testing. For our study, household HIV-positive results (based on the national testing algorithm) were confirmed by Geenius assay by a trained satellite lab technician. We observed slightly lower level of concordance (93.5%) between the algorithm and the Geenius assay. Therefore, WB was only conducted on the 131 samples that were positive by the national testing algorithm but negative on the Geenius assay. The WB indeterminate rate is not 74.8% (biased population). It identified additional 6 samples as positive by WB. Use of Geenius, and WB as needed, allowed us to calculate PPV using final results as the gold standard which otherwise would not be possible.

3. Line 75-76: authors state that Geenius is more feasible for use at the point of care. However, the interpretation of the bands by lay workers can be challenging and the use of a reader could help with this. However, the RDT reader is expensive and should be equipped with a software/laptop, which negates its use as a point-of-care, this should be mentioned.

Response: As rightfully pointed by your review, the Geenius assay is complex and cannot be performed by lay counselors/workers. The study had multiple testing points, namely household, satellite laboratory and central laboratory. The Geenius assay was performed at the satellite lab by trained lab technicians with an aid of automated reader and the software. We have updated the manuscript language to include the following*: The Geenius assay is a rapid cartridge format and has more feasibility of use at the near point of care, although it requires trained laboratory technicians and use of an automated reader for final interpretation (page 4, lines 77 to 79).*

4. Line 77–79: Before 2019, WHO recommended the use of a 2-test strategy in high prevalence settings and a 3-test strategy for low prevalence settings. As the study was conducted in 2018, it is important also to mention here the national HIV prevalence in Nigeria in 2018, and the rationale of using a 2-test strategy with a tie-breaker as part of the national HIV testing algorithm in a low prevalence country.

Response: Thank you for your important observation. Unfortunately, the NAIIS survey was restricted to implement the most current Nigeria’s national HIV testing algorithm. Our findings and publication of this peer reviewed article will help guide the necessary changes needed for Nigeria’s national program.

5. Line 97–88: please specify if Geenius HIV-1/2 RT were interpreted visually with the naked eye or with the aid of a Geenius reader with dedicated software.

Response: Thank you for the comment. The Geenius tests were performed by the Geenius reader and its dedicated software. We have added this information on page 5, lines 101 to 103; “*The Geenius HIV-1/2 RT assay was performed by trained laboratory technicians and the results were interpreted with an aid of an automated reader and its dedicated software provided by the manufacturer.”*

6. Line 150: the authors suggest that one likely explanation for high level of discordant results between T1 and T2 could be the low prevalence of HIV in Nigeria (1.4%). Whilst this is one factor that could explain this finding, and it is certainly the most likely explanation for the low PPV found in this study (94.5%), authors should also highlight that discordant results between T1 and T2 are generally expected given that T1 (Determine) is optimized for sensitivity (in order to rule in all positives) while T2 (Uni-gold) has generally a high specificity (in order to rule out false positives). Given this intrinsic small difference between individual tests (T1 and T2/T3), it would be useful to understand from the authors how they define this level of discordant results between T1 and T2 to be significantly high (i.e. 45%). How does this finding compare to other published data from other settings with a similar low HIV prevalence?

Response: Again, thanks for this observation and highlighting the importance of T1 and T2 discordance and overall low PPV. The population-based HIV-impact assessment (PHIA) surveys were conducted across multiple PEPFAR countries from 2014 to present. Our team is working on similar analysis looking at prevalence and overall PPV of the testing algorithms in Zimbabwe, Zambia, Malawi, Uganda, and Tanzania, to list a few countries. With higher burden countries, we observed higher concordance between T1/T2 and higher PPV of the testing algorithm. The draft manuscript from our findings is under development. We focused on Nigeria algorithm since we observed lowest T1/T2 concordance. Otherwise, the overall concordance ranged from 80% to 99.9%.

7. Line 153–154: given all the existing evidence showing that the use of a tie-breaker, to rule in HIV infection after discordant results between T1 and T2, can cause a high proportion of false positive diagnoses (Johnson CC et al, <http://dx.doi.org/10.7448/IAS.20.7.21755>), authors should elaborate further why this testing strategy continues to be used in Nigeria or at least make reference to the problem of misdiagnosis when employing a tiebreaker, this is not discussed at all in the manuscript.

Response: While I understand your argument, greater than 89% (64/72) of the specimens were confirmed as HIV-positive by the Geenius testing. Sharing additional breakdown of the 131 specimens that were not confirmed by geenius and potential false positive by national testing algorithm: T1/T2 pos = 123/131 and T1/T3 pos = 8/131. If T3 was not applied, greater than 43.5% of participants would have to be referred to additional testing in 4 weeks.

We agree that 3 consecutive reactive results should guide final HIV diagnosis in low prevalence country. This has been stated in the manuscript on Page 10, lines 172 to 174, “*As recommended by WHO, the use of a 3-test algorithm requiring consecutive three positive test results may be needed to achieve a PPV of 99.0% or higher when HIV prevalence is low.”*

8. Line 166–168: I appreciate this observation from the authors about the possible user-related errors that could have led to a high discordant rate between T1 and T2. However, the following sentence saying that all false positives were eventually identified by supplemental testing should be corrected to “most false positives were identified” as there was still 5.5% of individuals with positive diagnosis that could not be confirmed with supplemental testing, as stated in lines 147–148.

Response: Many thanks for suggesting the change. As suggested, this has been corrected in the manuscript. Page 10, line 173, *“…keeping in mind that most false positives were eventually identified by supplemental testing.”*

9. Line 172–174: authors list several practical implementation challenges of adding a 3rd test to the national testing algorithm including additional expenses to the national programme. However, a recent modelling study shows that the incremental cost of implementing a 3-test strategy vs a 2-test strategy is negligible because the 3rd test accounts for a small and diminishing share of total HIV tests (<https://www.medrxiv.org/content/10.1101/2021.03.31.21254700v1.full.pdf>). Authors should mention this evidence in their paper and weigh the costs of inadvertently initiating HIV-negative persons on lifelong ART as a consequence of misdiagnosis with the use of a two-test strategy, particularly in a low HIV prevalence setting such as Nigeria.

Response: Many thanks for sharing the publication (which may be under review and awaiting peer review clearance) and your recommendation on cost benefit on falsely putting people on treatment. We are just highlighting logistical and QA challenges of implementing the 3-testing strategy. Per your suggestion, we updated the language to the following: “*However, addition of a third test to the national algorithm can be challenging due to additional training and quality assurance requirements, procurement, inventory management, and budget implications to the national program.”* page 10, lines 181 to 182,

10. Line 181-183: given the high coverage of ART in many LMICs and globally overall, and the declining HIV positivity, authors should highlight that national HIV prevalence is no longer adequate to inform decisions about HIV testing strategies/algorithms and that treatment-adjusted prevalence is a more informative measure as described by Tippet Bett et al <https://www.ncbi.nlm.nih.gov/pmc/articles/PMC8640683/pdf/BLT.21.286388.pdf>

Response: Lines 181-183 described importance of retesting for verification, as recommended by WHO, which we believe is important to increase PPV of positive diagnosis. Last paragraph in the Discussion refers to reduced undiagnosed prevalence and need for a 3-test strategy to increase PPV. We have added suggested text to further highlight that national prevalence in no longer adequate to inform decisions about HIV testing strategies/algorithm (lines 175-178) citing the reference provided. Thank you for this suggestion.

Page 10, lines 175 to 178: “*Given the high coverage of ART in many LMICs and globally overall, and the declining HIV positivity, national HIV prevalence is no longer adequate to inform decisions about HIV testing strategies/algorithms and that treatment-adjusted prevalence is a more informative measure as described (Ref 11)*.”
